# Clinical MAPPs: a personalized healthcare-driven assay for the direct identification of potential T cell epitopes in patients

**DOI:** 10.3389/fimmu.2026.1879253

**Published:** 2026-07-07

**Authors:** Katharina Hartman, Guido Steiner, Cary M. Looney, Michel Siegel, Katharine Bray-French, Klaudia Brix, Sebastian Springer, Timothy P. Hickling, Niels Janssen, Axel Ducret, Céline Marban-Doran

**Affiliations:** 1 School of Science, Roche Pharma Research and Early Development, Roche Innovation Center Basel, F. Hoffmann-La Roche Ltd, Basel, Switzerland; 2 School of Science, Constructor University, Bremen, Germany

**Keywords:** anti-drug antibodies, dendritic cells, immunogenicity, MAPPs assay, mass spectrometry, personalized healthcare, T cell epitope, therapeutic antibodies

## Abstract

The development of anti-drug antibodies (ADAs) in response to therapeutic monoclonal antibody (mAb) treatments can undermine their efficacy and safety. A key player in this immunogenicity is the presentation of mAb-derived peptides by dendritic cells, which activates CD4+ T-helper cells. Traditionally, the MHC class II-associated peptide proteomics (MAPPs) assay is used preclinically to identify these peptides presented by monocyte-derived dendritic cells (moDCs). Here, we are introducing “clinical MAPPs”, an optimized, miniaturized version of the assay tailored for clinical use. Designed to work with cryopreserved peripheral blood mononuclear cells from low blood volumes (10 mL), clinical MAPPs offers a groundbreaking approach. Our proof-of-concept on patient material shows that clinical MAPPs enables personalized characterization of MHC-II receptor-associated antibody-derived peptides before mAb treatment. This innovative assay promises to be a critical tool for clinical immunogenicity risk assessments, enhancing personalized healthcare and ensuring safer, more effective treatments.

## Introduction

Therapeutic monoclonal antibodies (mAbs) have emerged as one of the largest classes of biologics (1) due to their desirable safety profile, superior efficacy, and ability to target specific epitopes with substantial specificity and potency (2, 3). This has revolutionized the treatment and management of various life-threatening and chronic diseases (3–5). Many of these drugs have set new standards of care, providing patients with a broader range of efficacious therapies (5, 6). Despite their clinical advantages, patients treated with mAbs may develop an unwanted humoral immune response against the drug in the form of anti-drug antibodies (ADAs), which is termed immunogenicity (5, 7–11). ADAs can bind to the active site of a mAb, preventing it from binding to its target antigen and potentially neutralizing its therapeutic effect. They can also accelerate the clearance of the drug, altering its pharmacokinetic (PK) profile and diminishing its pharmacodynamic (PD) effects. Beyond reducing efficacy, the presence of ADAs can lead to immunogenicity-related adverse events (AEs), thus impacting the safety of mAbs when administered to patients (12, 13).

Early prediction and mitigation of immunogenicity risks during early drug development are key for the development of safe and efficacious drugs (6, 10, 14, 15). Humanized and fully human sequenced-derived antibodies generally carry a lower risk for induced immune responses in humans compared to murine and chimeric antibodies (4, 9, 10, 16, 17), making them the dominant modalities in the field (18). However, the unique, non-germline peptide sequences in the complementarity-determining regions (CDRs) of these antibodies can nevertheless induce immunogenicity (10, 17). Furthermore, due to the multifactorial causes of immunogenicity, including treatment modality, product formulation, and patient-associated factors, achieving a goal of no immunogenicity may remain elusive, regardless of the degree of a mAbs’ optimization and humanization (9, 19).

The presentation by dendritic cells (DCs) of MHC-II receptor-bound antibody-derived peptides to CD4^+^ cells plays a pivotal role in the generation of a T-cell driven immune response. Accordingly, the sequencing and mapping of T cell epitopes is an essential step in assessing and mitigating immunogenicity risks (17). The major histocompatibility complex (MHC) class II-associated peptide proteomics (MAPPs) assay is a liquid chromatography tandem mass spectrometry (LC-MS/MS)-based immunogenicity risk assessment method that enables the identification and mapping of naturally processed and presented therapeutic mAb-derived peptides by monocyte-derived DCs (moDCs). Currently, however, MAPPs is exclusively used as an *in vitro* preclinical immunogenicity risk assessment tool (20, 21), mostly due to the assay’s requirement for high cell numbers that were only obtainable from healthy human blood donors. Thus, published results from clinical studies were achieved retrospectively probing therapeutic mAbs exhibiting known immunogenic risks in samples obtained from (unrelated) healthy human blood donors (21). Thus, a longitudinal, patient-specific correlation between the presentation of biotherapeutic-derived MHC-II peptides by DCs and the arising of ADAs has remained elusive to this date.

Herein we describe the development of a novel personalized healthcare tool, “Clinical MAPPs,” which enables the identification of naturally presented therapeutic mAbs-derived MHC-II peptides in individual patients enrolled in a clinical trial. We describe our semi-automated MAPPs’ sample preparation process, upstream of LC-MS/MS analysis to enhance the robustness of the assay. We also explain how this new protocol is designed to handle the lower cell numbers anticipated from a low volume blood collection (10 mL) and ensure compatibility with the use of frozen samples, while maintaining the functionality of the assay. Additionally, we compare the peptide presentation and activation profiles of moDCs, the cell type typically used in MAPPs, with their naturally occurring counterparts (DCs) to ensure their functional equivalence in the context of the MAPPs assay outcome. Finally, we demonstrate the feasibility of the proposed Clinical MAPPs protocol by comparing the MAPPs analysis of a biotherapeutic in both healthy individuals and patients enrolled in clinical trials. We believe that this personalized healthcare assay has great potential to facilitate the monitoring and management of ADA onset in patients both before and after treatment, thereby enabling the development of the most suitable treatment plan for each patient.

## Materials and methods

### Antibodies and compounds

The marketed therapeutic antibody adalimumab (Humira^®^, Abbvie, Chicago, IL, USA) was included as a clinical benchmark product purchased from a pharmacy and stored at 4 °C. The highly immunogenic antigen keyhole limpet hemocyanin (KLH-Imject Maleimide-Activated mcKLH; Thermo Scientific; Cat: #77600) served as a positive control. Stock solutions of KLH were reconstituted in sterile water at 10 mg/ml and stored at 4 °C. Adalimumab was used as a final concentration of 0.3 μM and KLH was used as a final concentration of 50 μg/mL.

### Human blood donors

Buffy-coat preparations (Blood Donation Center SRK, Aargau-Solothurn, Switzerland) and whole blood (F. Hoffmann-La Roche, Ltd, Basel, Switzerland) were obtained from consenting anonymous human healthy donors in accordance with current ethical practices. Cryopreserved peripheral blood mononuclear cells (PBMCs) from genotyped healthy human blood donors were provided by Lonza (Lonza Group AG; Basel, Switzerland), which were obtained from consenting anonymous donors in accordance with current ethical practices. Alternatively, PBMCs from selected patients enrolled in an ongoing clinical trial were collected prior to treatment after written informed consent was received in accordance with the Declaration of Helsinki. Approval was granted by the Institutional Review Board of Midlands Independent Review Board (Overland Park, KS, USA).

### Isolation of PBMCs from buffy coat or whole blood

Isolation of PBMCs from buffy-coats or whole blood was carried out via density gradient centrifugation using Ficoll-Paque PLUS (GE Healthcare; Cat: #17-1440-03) and SepMate tubes (Stemcell Technologies; Cat: #85460) according to the manufacturer’s recommendations under sterile conditions. Unless otherwise stated, fresh PBMCs were used for MAPPs and/or immunophenotyping. If using frozen samples, fresh PBMC aliquots were frozen in 10% (v/v) dimethyl sulfoxide (DMSO) in fetal bovine serum (FBS) at -80 °C overnight for a maximum of 72 hours and transferred to vapor-phased nitrogen at -196 °C.

### Direct isolation of human blood dendritic cells and characterization

Human circulating DCs were directly isolated from PBMCs according to the manufacturer’s instructions using the Blood Dendritic Cell Isolation Kit II for Humans (Miltenyi; Cat: #130-091-379). As moDCs and directly-isolated DCs were obtained from the same donor, anti-CD14 microbeads were first used to isolate monocytes to generate moDCs (as mentioned below) and the CD14 negative cell fraction (flow-through) was subsequently used to isolate blood DCs. All isolated blood DCs were cultured at a concentration of 0.3x10^6^ cells/mL in CellGenix DC medium (CellGenix; Cat: #20801-0500) supplemented with 1% (v/v) Glutamax (Gibco; Cat: #35050-061), 1% (v/v) non-essential amino acids (Gibco; Cat: #11140-035, 1% (v/v) sodium pyruvate (Gibco; Cat: #11360-039), 1% (v/v) penicillin-streptomycin (Gibco; Cat: #15140-122) in a humidified incubator at 37 °C with a physiological pH maintained in an environment of 5% CO_2_.

The purity and identification of PDC, MDC1, and MDC2 subsets was assessed by flow cytometry using the Dendritic Cell Enumeration Kit for Humans (Miltenyi: Cat: #130-091-086) with adaptations. In order to prevent unspecific binding of antibodies, Fc receptor (FcR) blocking reagent (Miltenyi: Catalog #130-059-901) was added to the samples prior to staining. PDCs, MDC1s, and MDC2s were detected by staining with the DC-specific antibodies CD303 (BDCA-2, FITC), CD1c (BDCA-1, PE), and CD141 (BDCA-3, APC), respectively, which was supplied as a pre-mixed cocktail in the kit. For exclusion of monocytes and B cells, additional samples were stained with CD14 (FITC, BD PharmingenTM: Catalog #557153) and CD19 (PE, BioLegend: Catalog #302208). Events were acquired on the BD LSR Fortessa and analyzed using the FlowJo software. Cells were gated based on their morphology (FSC/SSC), aggregation (FSC-H/FSC-A), and either expression of CD14/CD19 or DC subset-specific markers.

### Generation of monocyte-derived dendritic cells

Generation of moDCs from freshly-isolated (see above) or from frozen PBMC was performed using published protocols (22, 23) with the following modifications. Frozen PBMCs were rapidly thawed in a water bath at 37 ˚C and rinsed with a warm DC medium (as defined above). Cells were centrifuged twice (300 x g for 5 min at RT) and were re-suspended in 10–50 mL MACS buffer (Miltenyi; Cat: #130-091-221) final volume. Monocytes were isolated from PBMCs by positive immune-selection using anti-CD14 microbeads (Miltenyi; Cat: #130-050-201) on LS columns (Miltenyi; Cat: ## 130-042-401) and a QuadroMACS magnetic separator (Miltenyi; Cat: #130-090-976) following the manufacturer’s procedures under sterile conditions. Unless otherwise specified, 5.4x10^6^ (traditional MAPPs) or 2.5x10^6^-1.35x10^6^ (automated and clinical MAPPs) purified CD14^+^ mononuclear cells were cultured at a concentration of 0.3x10^6^ cells/mL in warm DC medium (as defined above). Monocytes were differentiated into immature moDCs using 5 ng/mL recombinant human IL-4 (R&D Systems; Cat: #204-IL) and 50 ng/mL recombinant human GM-CSF (R&D Systems; Cat: #215GM-500) for 5 days in a humidified incubator at 37 °C with a physiological pH maintained in an environment of 5% CO_2_. Cell counting was performed using a Bio Rad TC20 cell counter (Bio Rad; Cat: #1450102).

### DC activation assay

Following 12, 18, or 24 hours loading with 50 μg/mL KLH and activation with 1 μg/mL LPS of isolated moDCs and/or DCs from the same donor, DC activation markers were assessed by flow cytometry. To check for the differentiation of moDCs, monocytes, immature moDCs following differentiation, and stimulated moDCs for 24 hours were also subjected to the DC activation assay. FcR blocking reagent (Miltenyi; Catalog #130-059-901) was applied. Dead cells were stained using fix viability stain 510 (Biolegend; Cat: 564406). Extracellular markers were stained using antibodies against CD14 (M5E2, PerCP-Cy5.5), CD11c (B-Ly6, BV711), CD40 (5C3, BV786), B7-1/CD80 (L307.4, BUV737), CD83 (HB15e, APC), DC-SIGN/CD209 (DCN46, BV421), and CD86 (FUN-1, PE) from BD Biosciences and HLA-DR (L243, FITC) from Biolegend. Cells were gated based on their morphology, aggregation, viability, and expression of CD11c^+^, CD14^-^ for DCs and moDCs, and CD11c^+^, CD14^+^ for monocytes. For the differentiation evaluation, monocytes and (immature)moDCs were further analyzed according to their CD83, CD14 expression. For the moDCs versus DC activation time-course, the percent of parent for each activation marker/antibody is plotted as a 95% bootstrapped confidence interval.

### Loading, activation, and lysis of DCs and moDCs

DCs and moDCs were loaded with each individual test protein (0.3 μM individual mAb; 50 μg/mL maleimide-activated mcKLH, as specified), activated with 1 μg/mL lipopolysaccharide from Salmonella enterica (LPS; Sigma Aldrich; Cat: #L5886), and incubated for 24 hours (unless otherwise stated) at 37 °C and 5% CO_2_. Cells were harvested, washed, and cell pellets were lysed in for 1 hour in a hypotonic lysis buffer (20 mM Tris-HCl pH 7.8; 5 mM MgCl2) containing 1% (v/v) Triton X-100 (Roche Diagnostics GmbH; Cat: #11332481001) and a protease inhibitor mini tablet (Thermo Scientific; Cat: #A32955) in a ThermoMixer (Eppendorf; Cat: #5355000.011) at 1100 rpm and 4 °C. The lysates were collected following centrifugation at 14000 rpm for 10 min and 4 °C, and frozen at -80 °C prior to immunoprecipitation.

### MHC II-associated peptide proteomics assay

#### Manual immunoprecipitation protocol

The IP protocol was performed largely according to a published protocol (22). The mouse anti-human HLA-DR antibody (L243 clone, BD Pharmingen; Cat: #568232) was covalently coupled to N-hydroxysuccinimide (NHS)-activated sepharose based magnetic beads (GE Healthcare; Cat: #28-9537-64) as outlined by the manufacturer’s procedures and stored at 4 °C. Each cell lysate was incubated overnight with 50 μg (unless otherwise specified) equilibrated anti-HLA-DR-coupled magnetic sepharose beads at 4 °C on a rotator. The samples were washed three times each with buffer (20 mM HEPES pH 7.9; 150 mM KCl; 1 mM MgCl2; 0.2 mM CaCl2; 0.2 mM EDTA; 10% (v/v) glycerol; 0.1% (v/v) nonyl phenoxypolyethoxylethanol (NP)-40 alternative (Millipore; Cat: #492018-50ML)), with a wash buffer without NP-40, and with Milli-Q H_2_O. MHC-II peptides were eluted twice from HLA-DR molecules by adding 60 μL of 0.1% v/v trifluoracetic acid (TFA) for 30 min in the ThermoMixer (Eppendorf; Cat: 5355000.011) at 1100 rpm and 37 °C. The eluates were collected, evaporated in a SpeedVac at 30 °C (Thermo Scientific; Cat: #07-748-15), and kept at 4 °C until further use. Prior to mass spectrometric analysis, peptides were re-suspended in a 20 μL buffer containing 2% (v/v) acetonitrile and 0.5% (v/v) formic acid, and incubated in a ThermoMixer for 10 min at 1100 rpm and 37 °C. After centrifugation for 10 min at 30000 rpm, 17 μL of each peptide solutions were immediately transferred into mass spectrometry vials.

#### Automated immunoprecipitation protocol

The procedure was carried out largely according to a published protocol (23). Lysates were incubated either with 10 μg (unless otherwise specified) anti-human HLA-DR biotin (L243 clone, RayBiotech; Cat: #150-10306) or with a mixed preparation of 10 μg anti-human HLA-DR biotin (L243 clone, RayBiotech), 15 μg anti-human HLA-DQ biotin (clone SPV-L3, Biotium; Cat: #BNCB0200-500) and 15 μg anti-human MHC-II pan biotin (clone WR18; Bio-Techne, Cat: #NB100-64358B). The immunoprecipitation was performed overnight at 4 °C on a rotator, after which the preparations were transferred to a 96-well plate (Thermo Scientific; Cat: #AB1127). The isolation of the HLA receptors-peptides complexes was performed using the AssayMAP Bravo platform (Agilent Technologies, Cat: #3029078) and accompanying streptavidin cartridges (Agilent Technologies, Cat: #G5496-60021) as previously described (23). HLA II-specific peptides were eluted in 0.1% (v/v) TFA at a flow rate of 3 μL/min in a final volume of 20 μL in a low-binding 96-well PCR plate (Eppendorf; Cat: #0030129512). Peptides solutions were transferred to 0.5 mL low-binding PCR tubes Eppendorf; Cat #0030108094) and, after centrifugation for 10 min at 30000 rpm, 17 μL of each peptide solutions were immediately transferred into mass spectrometry vials. Alternatively, peptide samples were directly loaded onto Evosep C_18_ tips (Evosep Biosystems, Odense, Denmark; Cat: #EV2001) according to the manufacturer’s recommendations and stored at 4 °C until mass spectrometric analysis.

#### LC-MS/MS analysis of HLA-associated peptides

Eluted peptides were analyzed by liquid chromatography-electrospray ionization-tandem mass spectrometry (LC-ESI-MS/MS). The peptide mixture was separated using an UltiMateTM 3000 RSLCnano high performance liquid chromatography (HPLC) system with ProFlow technology (Thermo Scientific, Cat: #5200.0355) using an Aurora Elite column (15 cm x 75 μm i.d., 1.7 μm particle size, heated at 40 ˚C; Ion Opticks). Peptides were loaded for 2–3 minutes at 10 μL/min onto an Acclaim PepMap C_18_ trap column (100 μm i.d. × 20 mm, Thermo Scientific) using a vented-Tee design, followed by elution at a flow rate of 250 nL/min using a non-linear 39 min gradient of 2-45%B. The column was washed for 11 min and re-equilibrated for 10 min (buffer A: 0.1% (v/v) formic acid in 2% (v/v) acetonitrile/water; buffer B: 0.1% (v/v) formic acid in acetonitrile). Alternatively, peptide samples loaded onto EvoTips were separated using an Evosep One standardized nanoLC platform (Evosep, Odense, Denmark; EV-1000) using an Aurora Elite column (15 cm x 75 μm i.d., 1.7 μm particle size, heated at 40 ˚C; Ion Opticks). A turnaround time of 31 min was achieved using the Evosep’s built-in program 40 SPD Whisper. Either nanoLC devices were connected either to a Q-Exactive™ HF-X Orbitrap mass spectrometer (Thermo Scientific) or a trapped ion mobility time-of-flight (timsTOF) mass spectrometer (timsTOF PRO 2, Bruker) via a nano-electrospray interface. Using the Orbitrap mass spectrometer, eluted MHC II peptides were analyzed by data-directed analysis following standard operating parameters. Survey scans (scanning range m/z 400-1650) were recorded in the Orbitrap mass analyzer at a resolution of 60,000 with the lock mass option enabled. Data-dependent MS/MS spectra of the 12 most abundant ions from the survey scan were recorded in the Orbitrap cell at a resolution of 15,000 with dynamic exclusion set to 15 seconds. Using the TIMS TOF mass spectrometer, eluted MHC II peptides were analyzed by data-directed analysis following standard operating parameters. The TIMS accumulation/ramping time was set to 150 ms (mobility range: 0.6-1.6) while the TOF analyzer was set to record ions in the mass range m/z 100-1700 (global cycle time: 6.4 scans/sec). One survey scan (selection range for MS/MS analysis: m/z 350-1300, ion mobility 0.7-1.5, 1<z<6) was followed by MS/MS analysis in PASEF mode including up to 10 TIMS ramps per full cycle. Dynamic exclusion prevented the repeated selection of an ion for MS/MS analysis for 9 sec.

#### Data analysis

Raw mass spectrometric data were searched with PEAKS Xpro Studio software (Bioinformatics Solutions Inc.) against the human protein database UniProtKB (http://www.uniprot.org, release 2015_10, 88500 TrEMBL and SwissProt entries containing the amino acid sequences of the test therapeutic proteins). Searches were performed with a tolerance of 15.0 ppm/10.0 ppm (TIMS-TOF/Orbitrap precursor mass) and 0.05/0.03 Da (TIMS-TOF/Orbitrap fragment ions) using the unspecific digest mode. Met-sulfoxide, Asn/Gln de-amidation and N-terminal pyro-glutamylation were considered as differential modifications. Batch-wise processing was performed for all LC-MS/MS runs of a given donor. Peptide results were filtered at 1% false discovery rate cutoff, and the corresponding PSM result table was exported in a tab-delimited table without further normalization. Heat maps and accompanying diagrams were generated with dataMAPPs - an in-house developed R-based workflow for the quality control, processing, and analysis of MAPPs data (22).

The binding of identified peptides to HLA-DRB receptors was analyzed using the NetMHCIIpan-4.2 server (https://services.healthtech.dtu.dk/services/NetMHCIIpan-4.2/) with recommended settings (24).

## Results

### A conventional MAPPs assay identifies the potential T cell epitopes from adalimumab presented on moDCs

Adalimumab (Humira^®^) is a fully human mAb that binds to and suppresses tumor necrosis factor (TNF)-α. It is approved for the treatment of various conditions, including rheumatoid arthritis, psoriatic arthritis, and Crohn’s disease (18, 25, 26). It was selected as a suitable therapeutic mAb for benchmarking due to readily accessible sequence information that would aid in the analysis of the peptidic response pattern. Additionally, extensive research in the literature addresses its immunogenic potential, particularly regarding the ADA response rate in patients (18, 27–31) and previously identified T cell epitopes (32). Adalimumab is available in Good Manufacturing Process (GMP) quality, ensuring the homogeneity and consistency of the protein preparation.

**Figure 1:**
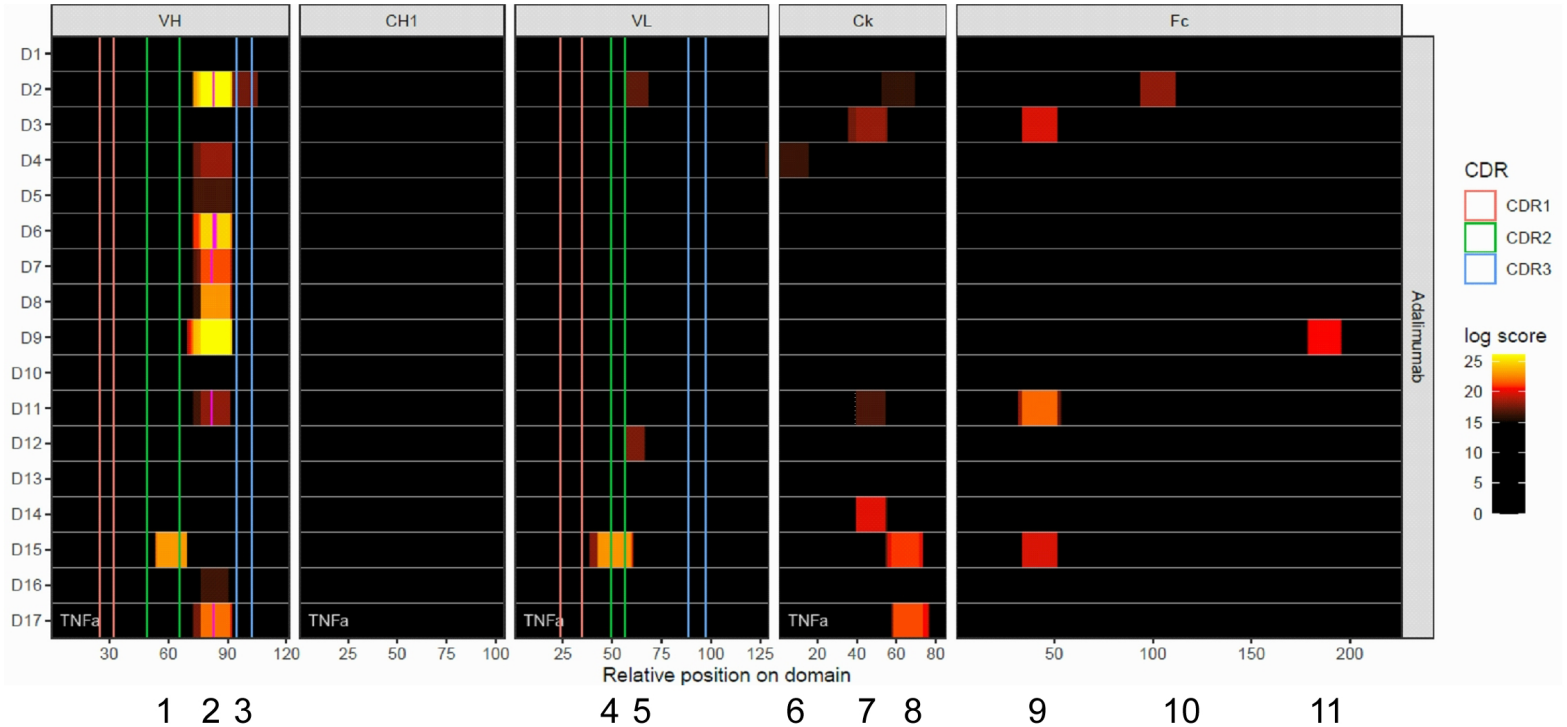
Heat map of HLA-DR-associated adalimumab peptides presented by moDCs from 17 healthy buffy-coat donors. HLA-DR-associated peptides were immunoprecipitated from 5.4x10^6^ adalimumab-loaded moDCs and eluted peptides were identified by LC-MS/MS using the QE-HFX mass spectrometer. The adalimumab sequence regions are separated by the variable domain of the heavy chain (VH), constant domain of the heavy chain (CH1), variable domain of the light chain (VL), constant region of the kappa type light chain (Ck), and the fragment crystallizable (Fc) region. Identified clusters are signified by the colored regions spanning from dark red to yellow, which represent the antibody peptide abundance (as a log2 score) per position from low to high, respectively (indicated in the legend). Complementarity-determining regions (CDRs) 1 to 3 are indicated as pink, green, and blue vertical lines, respectively, along the sequence of the VH and VL domains. Identified peptide clusters are indicated as C1 to C11 below the horizontal axis of the relative adalimumab amino acid sequence position (see **Supplementary Table 1**). Donor number (D1-D17) is denoted on the vertical axis. Vertical pink lines denote a modified amino acid (here oxidized VH Met_86_). The data was generated using a Q-Exactive™ HF-X Orbitrap mass spectrometer.

We initially performed the previously described “conventional” MAPPs assay (22) using moDCs as the professional APCs, differentiated from monocytes isolated from PBMCs obtained from healthy human donors. Immature moDCs were challenged with adalimumab and activated with lipopolysaccharide (LPS) for 24 hours, after which they were lysed and the protein extracts were incubated overnight with the anti-human HLA-DR antibody-coupled NHS-activated sepharose-magnetic beads. After immunoprecipitation, eluted MHC-II peptides were subsequently determined via LC-MS/MS analysis. The mapping of HLA-DR receptor-specific peptides, which can originate from different sequence regions of a protein and usually occur in multiple length variants (33), are depicted in a heat map of potential T cell epitopes from adalimumab (**Figure 1**). A peptide “cluster” consists of overlapping peptides containing a common HLA-DR binding core region. Within a cluster, abundance is indicated using distinct color codes by summing the abundance of common amino acids of overlapping MHC-II peptides.

From the 17 healthy buffy-coat donors tested, a total of 11 adalimumab-derived peptide clusters are identifiable (**Supplementary Table 1**), spanning the variable heavy (VH) domain, variable light (VL) domain, constant light (CL) domain, and crystallizable fragment (Fc) regions. 10 donors presented clusters derived from the VH domain (clusters 1-3) with Donors 2 and 15 presenting an additional cluster of peptides derived from CDR3 or CDR2, respectively. Only Donor 15 presented a cluster from the VL region (cluster 4), which overlapped with the CDR2 region. 3 of the 17 donors (Donors 1, 10, and 13) did not present any adalimumab-derived peptides. The sequences in the variable regions of adalimumab that have the potential to induce T cell responses, such as clusters 1, 2, 3, and 4, were reported by Meunier et al (32). and Sekiguchi et al (34). Overall, the results indicate that adalimumab-derived peptide clusters can be identified via the traditional MAPPs assay protocol, making it a suitable therapeutic mAb to use as a benchmark for the development of Clinical MAPPs.

### An automated MAPPs sample preparation protocol increases the identification of HLA-DR receptor-specific peptides at lower moDCs counts and enables the use of cryopreserved PBMCs

**Figure 2:**
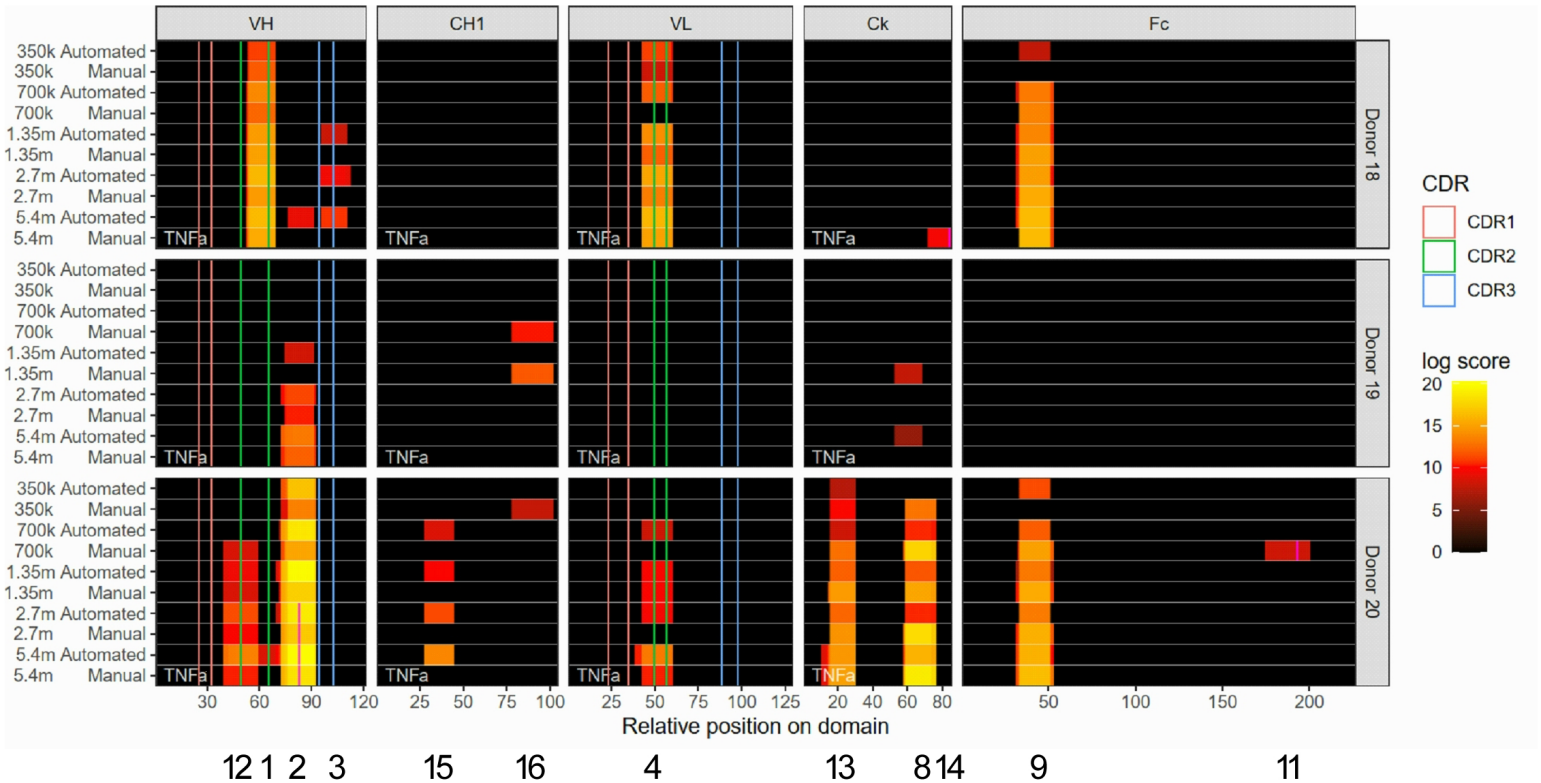
An automated sample preparation protocol leads to the presentation of additional adalimumab-derived peptide clusters overlapping with CDRs in the MAPPs assay. Comparative heat map of HLA-DR-associated adalimumab-derived peptides presented by differing numbers (3.5x10^5^, 7.0x10^5^, 1.35x10^6^, 2.7x10^6^, 5.4x10^6^) moDCs from the same buffy-coat donor. Eluted peptides from both preparation types were identified by LC-MS/MS using the Bruker TIMS ToF mass spectrometer. The adalimumab sequence regions are separated by the variable domain of the heavy chain (VH) and light chain (VL), constant domain of the heavy chain (CH1), constant region of the kappa type light chain (Ck), and the fragment crystallizable (Fc) region. Clusters are signified by the colored regions spanning from dark red to yellow, which represent the antibody peptide abundance (as a log2 score) per position from low to high, respectively (indicated in the legend). Complementarity-determining regions (CDRs) 1 to 3 are indicated as pink, green, and blue vertical lines, respectively, along the sequence of the VH and VL domains. Identified clusters are indicated as C1 to C16 below the horizontal axis of the relative amino acid sequence corresponding to each domain (see **Supplementary Table 1**). Donor number, protocol used (Bravo vs manual), and cell number of each sample is denoted on the vertical axis. Identified peptide clusters in relation to the clusters identified in **Figure 1** are indicated below the horizontal axis of the relative adalimumab amino acid sequence position. Data obtained from 3 buffy-coat donors. Vertical pink lines denote a modified amino acid. The data was generated using a TIMS ToF Pro2 mass spectrometer.

High quality, reproducible MAPPs assay results depend on a precise and consistent sample handling (35). A robust and reproducible protocol is particularly critical for clinical MAPPs assays, which must operate with reduced cell numbers due to the limitations in blood volumes that patients can provide during hospital treatments. The conventional MAPPs assay (as described above) requires manual coupling of the HLA-DR antibody to magnetic sepharose beads, manual IP of HLA-DR receptor-specific peptide complexes and manually eluting the peptides. In addition to potential operational errors and inter-operator variability, this workflow is labor-intensive, time-consuming, and prone to operational errors and inter-operator variability. To address these challenges, we have recently adapted the sample preparation protocol to run on an automated, high-throughput, sensitive, and reproducible IP and elution platform. This robotic platform processes samples in a 96-well plate format using streptavidin resin-containing cartridges to capture biotin-conjugated anti-HLA antibody when processing cell lysates (23). Depending on the input sample volume (which is proportional to the input cell number) and flow rate, the automated protocol can process up to 96 samples simultaneously within about 2 hours, with 1 hour of additional manual intervention required for setup and buffer preparation. This represents a substantial improvement over the manual protocol, which initially took 3 days and could only accommodate 24 samples simultaneously. We therefore investigated the sensitivity and reproducibility of the manual versus automated MAPPs protocols in three donors, with starting cell inputs ranging from 350–000 to 5.4 million moDCs-derived CD14^+^ monocytes (the number of cells used in a traditional MAPPs assay; **Figure 2**; a detailed output for this analysis can be found in **Supplementary Table 2**). At each input cell number tested, the automated protocol consistently identified a higher number of total unique peptides across all donors compared to the manual protocol even when using the same (more sensitive) mass spectrometer.

An additional requirement in using MAPPs in a clinical setting is the necessity of using frozen PBMCs as starting material since blood samples are typically processed locally and PBMCs are usually stored frozen prior to shipping for subsequent analysis. Although studies have concluded that phenotypic and functional assays can be performed with frozen PBMCs instead of fresh ones (36), cryopreservation has also been shown to negatively affect cell viability, phenotype, and cellular functionality (37). Therefore, we investigated the feasibility of using cryopreserved PBMCs isolated from 10 mL of whole blood (a volume typically collected in clinical settings) in the MAPPs assay. We compared the monocyte isolation recoveries (**Supplementary Figure 1**) and KLH-derived peptide cluster profiles obtained from fresh versus frozen PBMCs isolated from 10 donors. KLH was chosen as a technical control to ensure a strong immune response, enabling a qualitative comparison of freshly isolated and frozen PBMCs in the MAPPs assay. All other experimental factors were kept constant, including the number of CD14^+^ monocytes used for each condition prior to immunoprecipitation.

**Figure 3:**
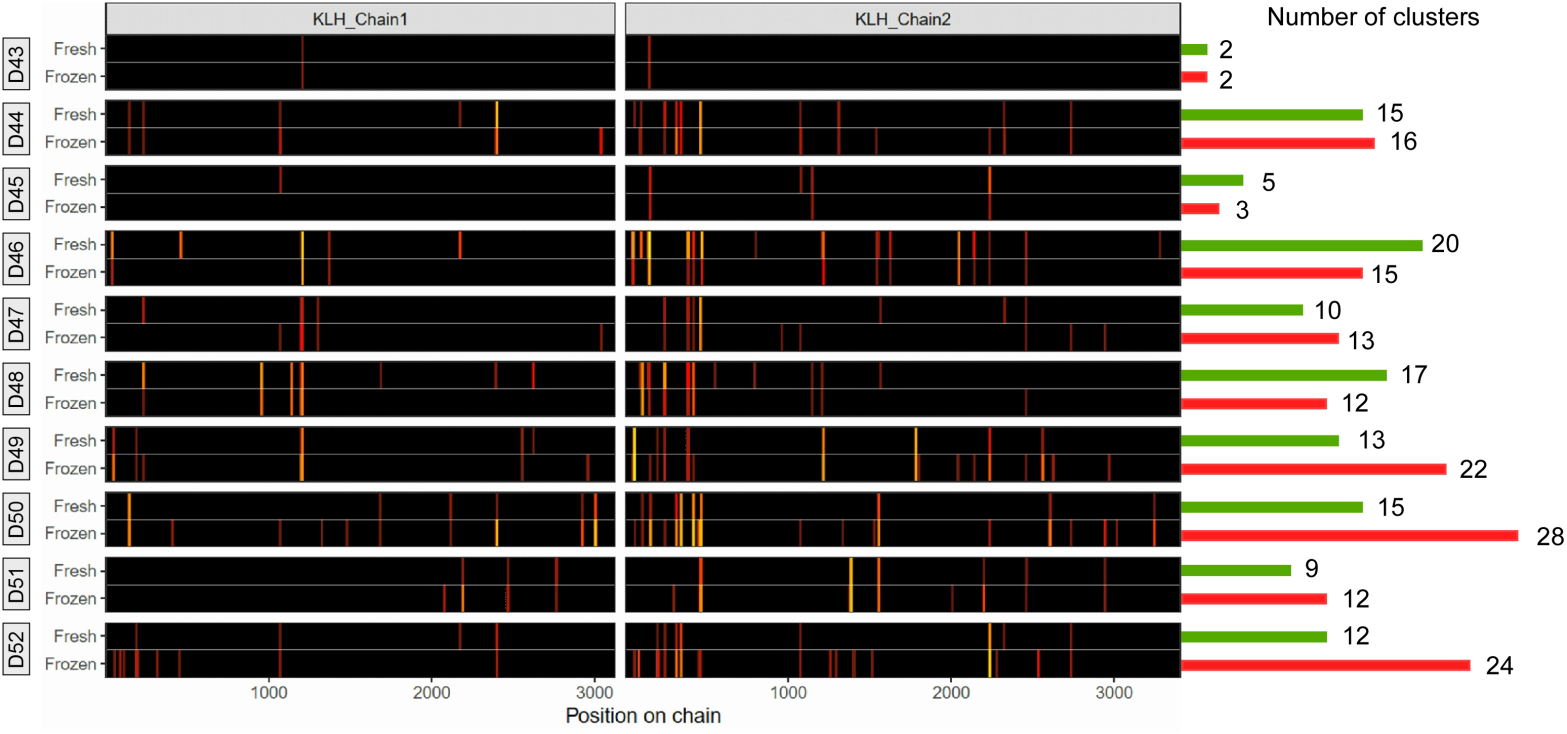
Applying the clinical MAPPs assay protocol to be set forth using cryopreserved PBMCs in comparison to fresh PBMCs. Cryopreserved and fresh PBMCs each obtained from 10 mL whole blood per donor were subjected to the MAPPs assay. moDCs were challenged with KLH and HLA-DR-associated peptides were immunoprecipitated and identified by LC-MS/MS using the TIMS ToF mass spectrometer. Identified clusters are signified by the colored regions spanning from dark red to yellow, which represent the abundance of KLH-derived peptides from low to high, respectively. Donor number and starting material (fresh or frozen PBMCs) is denoted on the vertical axis. Data obtained from 10 whole blood donors. Starting number of CD14^+^ monocytes: D43, 2.63x10^5^ cells; D44, 1.97x10^5^ cells; D45, 2.40x10^5^ cells; D46, 2.08x10^5^ cells; D47, 1.26x10^5^ cells; D48, 4.48x10^5^ cells; D49, 1.86x10^5^ cells; D50, 2.13x10^5^ cells; D51, 1.09x10^5^ cells; D52, 3.01x10^5^ cells. All data were generated using a TIMS-ToF Pro2 mass spectrometer.

The MAPPs analysis of moDCs isolated from 10 HLA-typed blood donors (comparing fresh versus frozen PBMCs as starting material) led to donor-specific, highly comparable KLH cluster profiles and peptide abundances (**Figure 3**; detailed outputs of this analysis can be found in **Supplementary Table 3**; **Supplementary Figure 2**). Overall, peptide yields from DC preparations isolated from frozen PBMCs were somewhat lower than from preparations isolated from fresh PBMCs (75% ± 25%). For three donors (D44, D50, and D51), the number of isolated peptides was comparable between fresh and frozen PBMC-derived moDCs; conversely, frozen preparations from two donors (D45 and D46) yielded a significantly lower number of peptides (**Supplementary Table 3**). In this experiment, there was no direct correlation between the number of CD14^+^ monocytes used in each preparation and the number of unique peptides that were identified in the MAPPs assay, possibly due to cell death during differentiation and antigen challenge (typically, 50 to 90%, **Supplementary Figure 1**). However, most importantly, the peptide length distribution in both experimental sets was highly characteristic of a highly enriched MHC-II peptide preparation, with a maximum of around 15–16 amino acids in length (**Supplementary Figure 2A**). Interestingly, at an equivalent number of total peptides identified, the number of KLH-derived peptides and clusters were usually greater in moDCs prepared from frozen PBMCs, for example, in donors D44, D47, and D49-D52. In these last four donors, KLH-derived peptide abundance also appeared to be higher when using frozen PBMCs as the source material. We investigated the binding specificity of endogenous and KLH-derived MHC-II peptides obtained from both fresh and frozen cohorts for each donor to HLA-DRB1/3/4/5 alleles using the NetMHCIIpan-4.2 software package (24) (**Supplementary Table 3**; **Supplementary Figure 2B**). The analysis demonstrated that the MAPPs process, using a pan-HLA DR immunoprecipitating antibody, strongly enriches for HLA-DR receptor-binding peptides regardless of whether the starting material was fresh (mean 83.8% of total unique peptides, stdev 8.5%) or cryopreserved (mean 83.5% of total unique peptides, stdev 6.8%) PBMCs. Furthermore, a large majority of the HLA-DR-specific peptide binders identified from frozen PBMCs overlapped with binders identified from fresh PBMCs (**Supplementary Table 3**), indicating that the immunopeptidomes of frozen PBMCs are consistent with those of fresh PBMCs.

### Donor-specific moDCs and circulating DCs exhibit comparable peptide presentation and activation profiles

DCs represent an extremely rare mononuclear cell type in blood, accounting for only about 1% of PBMCs (38), in contrast to moDCs, which are differentiated from the much more abundant CD14^+^ monocytes (about 15% of PBMCs) (39). Consequently, the use of moDCs has become a more viable alternative to run a MAPPs assay, especially when blood volume is limiting. However, to our knowledge, the equivalence in peptide processing and presentation between circulating DCs and moDCs in the MAPPs assay has not been thoroughly investigated or reported. To address this, we prepared moDCs and circulating DCs from the same healthy donors to perform a side-by-side comparison, focusing on purity after isolation, differentiation, response to LPS stimulation and MHC-II peptide processing and presentation.

**Figure 4:**
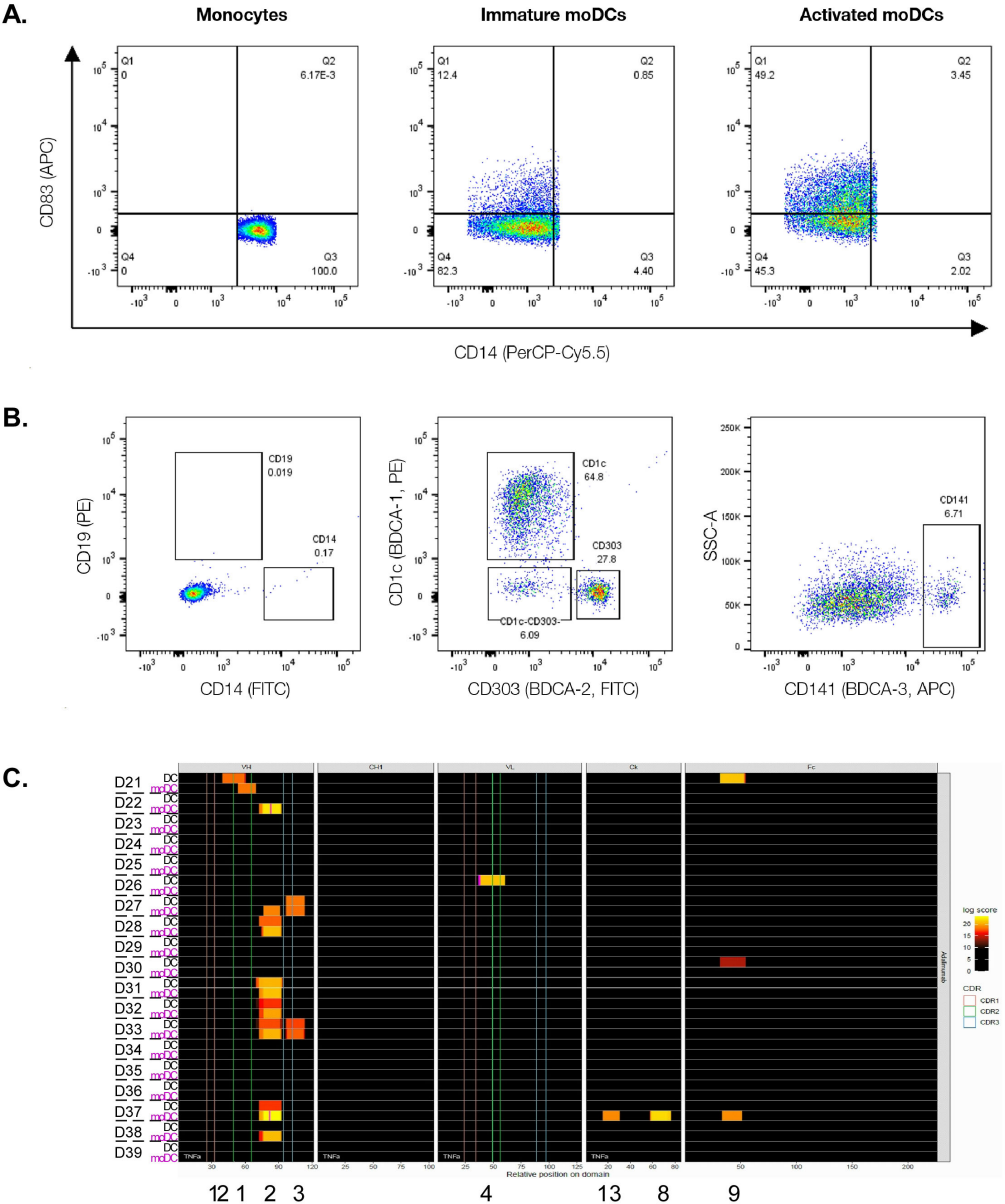
moDCs and DCs present similar phenotypic characteristics when used in the MAPPs assay **(A)** Flow cytometry analysis comparing monocytes with immature and stimulated moDC. Shown are the CD83 and CD14 surface marker expression identified from the parent CD11c^+^CD14 population. Data is representative of 2 buffy-coat donors. **(B)** Flow cytometry analysis demonstrative of highly enriched, sorted DCs. *Left panel*: the low signals in the gated CD14 and CD19 positive cells, for the identification of monocytes and B cells, highlight a homogenous parent DC population. *Middle and right panels:* Plasmacytoid DC (PDC), myeloid DC type 1 (MDC1), and MDC2 subsets are identified via CD303, CD1c, and CD141, respectively. The numbers correspond to the percentage of cells within each respective gate. Data is representative of 2 buffy-coat donors. **(C)** Heat map of human HLA-DR-associated adalimumab peptides presented by moDCs and naturally existing blood DCs isolated from the same donor using 19 healthy buffy-coats. Eluted peptides were identified by LC-MS/MS using the Bruker TIMS ToF mass spectrometer. Identified peptide clusters are indicated below the horizontal axis of the relative adalimumab amino acid sequence position, and numbered relative to the clusters identified in **Figures 1** and **2**. The number of moDCs and DCs were adjusted to the same cell number: D21, 6.83x10^5^cells; D22, 5.46x10^5^cells; D23, 8.93x10^5^cells; D24, 3.60x10^5^cells; D25, 6.00x10^5^cells; D26, 6.83x10^5^cells; D27, 8.57x10^5^cells; D28, 9.83x10^5^cells; D29, 8.79x10^5^cells; D30, 4.00x10^5^cells; D31, 6.00x10^5^cells; D32, 10.00x10^5^cells; D33, 5.50x10^5^cells; D34, 6.03x10^5^cells; D35, 5.82x10^5^cells; D36, 7.00x10^5^cells; D37, 14.00x10^5^cells; D38, 10.00x10^5^cells; D39, 27.00x10^5^cells. The data was generated using a TIMS ToF Pro2 mass spectrometer.

First, we characterized the differentiation of monocytes into immature moDCs and their maturation upon stimulation to monitor their functionality. The expression levels of the CD14 monocyte marker and the DC activation marker CD83 from CD11c^+^ parent populations were used to evaluate purity after isolation, differentiation and response to LPS stimulation (**Figure 4A**). Upon differentiation, monocytes lost expression of the CD14 marker while LPS stimulation increased the level of the CD83 marker, indicating a successful differentiation and stimulation of moDCs (40). To explore whether DCs directly isolated from blood can be used in a MAPPs assay, we employed a two-step magnetic separation protocol to isolate distinct human DC subtypes: myeloid DC type 1 (MDC1), myeloid DC type 2 (MDC2), and plasmacytoid DC (PDC). The non-DC blood mononuclear cells (CD14 and CD19 cells) were first depleted via negative selection, followed by positive selection with blood DC antigens CD303, CD1c and CD141 to obtain highly enriched circulating DCs. Following isolation, the purity and identification of human DC subtypes was assessed using flow cytometry. The purity of circulating blood DCs exceeded 99%, with the main contaminants being CD14^+^ monocytes (0.17%) and CD19+ B cells (0.019%) (**Figure 4B**). Among all circulating DCs, approximately 64.8% were CD1c^+^CD303^-^ MDC1s, 27.8% were CD1c^-^CD303^+^ PDCs, and 6.71% were CD141^+^ MDC2s. This is in agreement with literature values of ~60% MDC1, ~37% PDC, and ~3% MDC2 (41–43). We then investigated whether circulating DCs could be directly loaded with an antigen of choice and subsequently analyzed using the MAPPs assay. Specifically, we aimed to compare the MAPPs profiles obtained from DCs and moDCs from the same donor. To maximize the DC yield, we first isolated the CD14^+^ monocytes fraction and then subsequently used the CD14^-^ fraction to isolate circulating DCs. Across 29 donors, an average of 0.46% (2.44 million cells) DCs and 15.39% (81 million cells) monocytes were recovered from PBMCs from the same buffy-coat donor (**Supplementary Figure 3**). In a direct side-by-side comparison, using an identical number of cells in the MAPPs assay, circulating DCs and moDCs from the same donor exhibited a similar though not absolutely identical MHC-II peptide profile when challenged with adalimumab (**Figure 4C**). For 5 donors, moDCs presented peptide clusters not presented by DCs from the same donor, while the reverse was true for three donors (1 of these 3 donors were also part of the initial six mentioned above). For instance, in Donor 1 and 27, only the DCs and not the moDCS presented cluster 9, while this same cluster was presented exclusively by moDCs but not by DCs in Donor 38.

**Figure 5:**
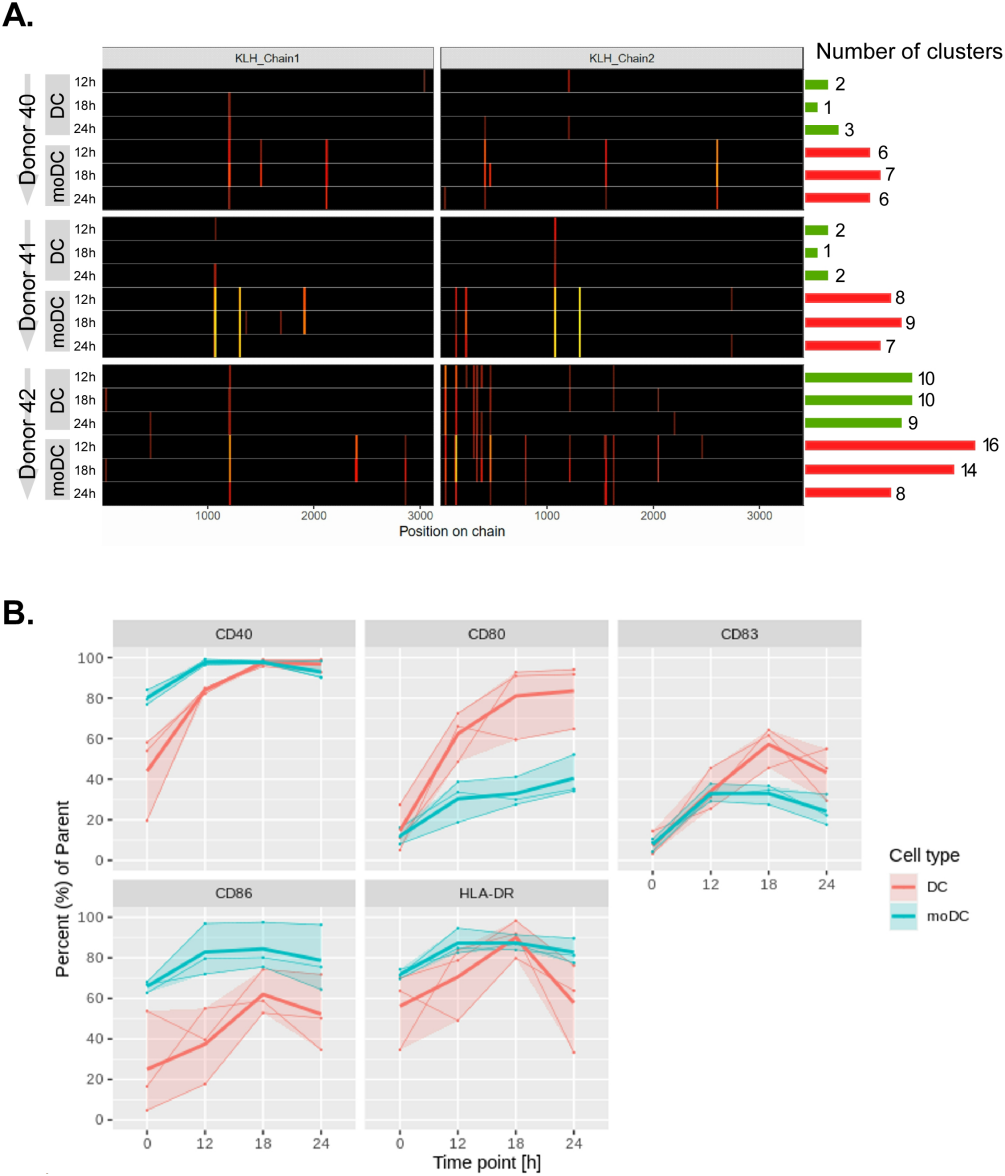
Dendritic cell versus monocyte-derived dendritic cell (moDC) peptide presentation and activation time-course. **(A)** Heat map of HLA-DR-associated keyhole limpet hemocyanin (KLH) peptides in the MAPPs assay. HLA-DR-associated peptides were immunoprecipitated and eluted peptides were identified byLC-MS/MS using the TIMS ToF Pro2 mass spectrometer. Identified clusters are signified by the colored regions spanning from dark red to yellow, which represent the abundance of KLH-derived peptides from low to high, respectively. Donor number, cell type (moDC or DC), and stimulation time-point is denoted on the vertical axis. The number of moDCs and DCs were adjusted to the same cell number: donor 40, 2.17x10^5^ cells; donor 41, 3.54 x10^5^ cells; donor 42, 2.22 x10^5^ cells **(B)** 95% bootstrapped confidence intervals of the percent of parent for the surface expression of each DC activation marker (CD40, CD80, CD83, CD86, and HLA-DR). Individual donors are denoted as thin lines, mean values as bold lines, and confidence intervals as transparent colored areas. moDCs or DCs were isolated from the same donor using 3 healthy buffy-coats, and both cell types were treated and stimulated with KLH and lipopolysaccharide (LPS) for either 12, 18, and 24 hours.

The minor discrepancies observed in the MAPPs experiment when comparing the peptide presentation profiles between moDCs and DCs prompted us to investigate whether these differences were primarily due to biological or technical factors. To address this, we conducted a time-course activation experiment with three donors to determine whether the activation state of moDCs and circulating DCs, as measured by flow cytometry, correlated with peptide presentation via MAPPs in response to LPS stimulation and KLH challenge (KLH was used as a technical positive control in the DC activation assay (44) and the MAPPs assay (22)). In the context of the MAPPs assay, an 18 to 24 hours stimulation period resulted in the most optimal HLA-DR-specific KLH-derived peptide presentation for both cell types (**Figure 5A**, e.g. donors 40 and 41). Interestingly, for donor 42, a shorter stimulation period of 12 to 18 hours was preferred over the longer 24-hour time point. Notably, the KLH-derived MHC-II peptide profiles obtained from DCs and moDCs were highly consistent within each donor, regardless of the stimulation duration, which primarily influenced the abundance of individual clusters. In general, moDCs consistently present more peptides, possibly due to a higher abundance of MHC-II peptide receptors on this cell type. In the DC activation assay, both DCs and moDCs stimulated with LPS for either 18 or 24 hours exhibited an activated phenotype, characterized by a high surface expression of CD40 (co-stimulation for T and B cell activation), CD80 (co-stimulation for T cell activation), CD83 (lymphocyte activation), CD86 (co-stimulation for T cell activation), and HLA-DR (antigen presentation (21)) (**Figure 5B**). Interestingly, the data indicate that the highest HLA-DR surface expression in DCs was achieved after 18 hours of stimulation, while moDCs showed no substantial changes in HLA-DR expression between 12 to 24 hours of stimulation. Conversely, the maximal surface expression levels for CD83 and CD86 were reached after 18 hours stimulation for both cell types. Since the highest HLA-DR receptor expression should directly correlate with optimal MHC-II peptide presentation in the MAPPs assay, we conclude that an 18-hour stimulation is most ideal for DCs and moDCs in our current assay conditions. Taken together, these results suggest that *in vitro* derived moDCs exhibit comparable activation profiles and an enhanced peptide presentation compared to circulating DCs, thereby confirming that moDCs are a valid proxy for monitoring MHC-II receptor peptide presentation in the MAPPs assay.

### Clinical MAPPs enables the analysis of patient-derived samples supporting a personalized immunogenicity risk assessment

In the previous sections, we outlined key improvements in our sample preparation method and analytical procedure to significantly improve the sensitivity and robustness of the MAPPs assay. In parallel, we demonstrated that cryopreserved PBMCs are a suitable source of biological material for generating moDCs. Additionally, we established that moDCs serve as an acceptable proxy for naturally occurring DCs in the context of the MAPPs assay. With these advancements, we felt confident to initiate a proof-of-concept study aimed at demonstrating the utility of this approach for characterizing naturally processed and presented MHC-II peptides in patients enrolled in a clinical trial. For this purpose, we obtained cryopreserved PBMCs (isolated at baseline from 10 mL of whole blood) from 43 donors enrolled in a clinical trial. We subsequently challenged the patients’ moDCs with the biotherapeutic before conducting the MAPPs assay. As a comparator, we used cryopreserved PBMCs obtained from 30 healthy donors, following the same protocol except for using a fixed number of moDCs (1.35x10^6^ cells) to run the assay (**Supplementary Table 4**). Given our recent publication on the potential benefits of a panHLA approach to monitor additionally HLA-DP and -DQ peptide-receptor complexes, we investigated whether such an approach could be useful in a clinical setting and whether a combined immunoprecipitation approach (combining three antibodies to enrich for HLA-DR, -DP, and -DQ receptor complexes (23)) may trigger additional findings.

**Figure 6:**
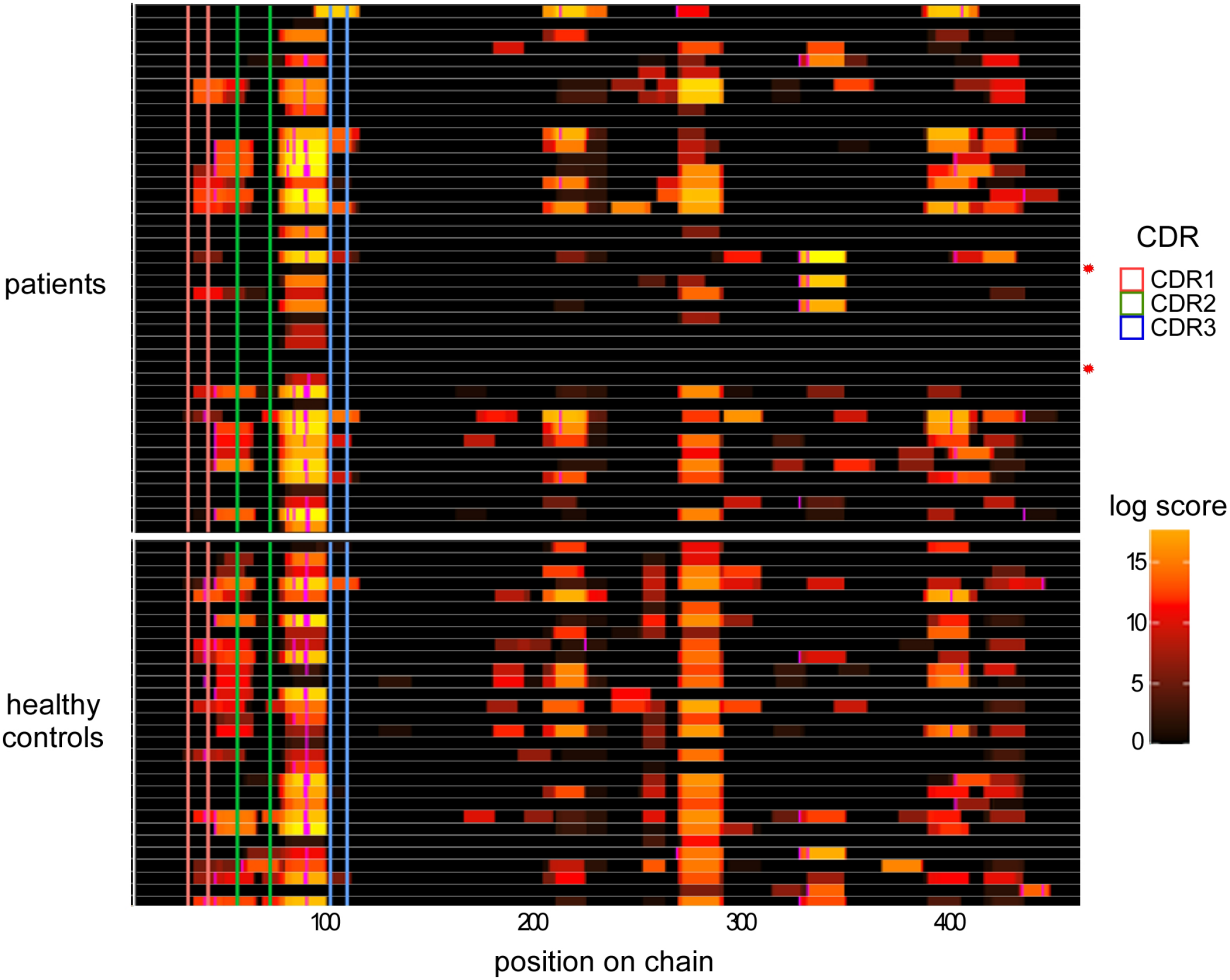
Applying the clinical MAPPs assay protocol using cryopreserved PBMCs either from 43 patients enrolled in a clinical trial (upper panel) or from 30 healthy control donors (lower panel). The heavy chain of the biotherapeutic’s amino acid sequences is represented with the positions of the three hypervariable regions (CDR1, 2 and 3) indicated by the colored vertical lines. The PBMCs obtained from patients enrolled in a clinical trials were isolated from 10 mL whole blood prior to immediate cryopreservation. After thawing, CD14^+^ monocytes were isolated and differentiated to immature moDCs, which were challenged with the biotherapeutic and matured with LPS prior to the MAPPs assay. The same protocol was used to analyze cryopreserved PBMCs obtained from a healthy control donor cohort. Heat map of HLA-DR-associated keyhole limpet hemocyanin (KLH) peptides in the MAPPs assay. HLA-DR, DP and DQ-associated peptides were immunoprecipitated and eluted peptides were identified by LC-MS/MS using the TIMS ToF mass spectrometer For analysis, 1.35x10^6^ monocytes were used for healthy controls while there was no cell number adjustment for patient’s samples (i.e., 100% of the obtained monocytes were used for the assay – see **Supplementary Table 4** for detail). The stars denote samples for which there were no compound-related results obtained in the MAPPs assay. All data were collected using a TIMS ToF Pro2 mass spectrometer.

The peptide presentation profile of the heavy chain of the biotherapeutic in patients and healthy volunteers is shown in **Figure 6**. The MHC-II peptide profile obtained is remarkably similar between patients and healthy donors, both in terms of the positions of MHC-II clusters and their abundance despite the HLA variability within the two cohorts. As reported previously (23), the use of a panHLA immunoprecipitation strategy led to the detection of additional MHC-II peptide clusters, although most of these were specific to HLA-DR receptors (data not shown). Encouragingly, the MAPPs assay successfully profiled clinical patients using a starting volume of 10 mL of whole blood, which can be routinely collected in clinical settings. However, results were not obtained from five of the 43 clinical donors, mainly because of a low yield of moDCs or low PBMC counts (**Supplementary Table 4**). Given the very limited number of cells available, we opted to process all samples using a single condition to maximize yield of MHC-II peptides (1.35x10e6 CD14+ cells). Consequently, an assessment of moDCs viability and fitness, typically assessed using KLH as positive control, could not be performed in this setup.

## Discussion

A persistent challenge in the development of innovative biotherapeutics, particularly these that have been designed to exhibit novel biological activities, is the potential for unwanted immune responses (reviewed in (45)). Specifically, a comprehensive assessment of the likelihood of ADA formation, their impact on efficacy, PK/PD and patient safety, along with the implementation of appropriate risk mitigation strategies, has become an essential component of biotherapeutic development plans and is mandated by regulatory agencies (46, 47). One empirical approach to reducing immunogenicity risks involves minimizing or avoiding the incorporation of non-human components in antibody design. While this approach is generally advantageous, merely using a fully human amino acid sequence alone does not inherently eliminate the risk of ADA formation (48). Over the past decade, an increasing number of *in silico*, *in vitro*, and *in vivo* assays have been used during pre-clinical stages to aid in selecting the best clinical candidate. These assays, combined with other selection criteria, such as *in vitro* potency, *in vivo* efficacy, PK/PD characteristics and safety, help identify the optimal molecule with the lowest risk of immunogenicity (20, 21, 49). Demonstrating proof-of-concept for mitigating immunogenicity risks has been a challenging and complex endeavor, as directly testing the causal relationship between an identified immunogenicity risk and the clinical occurrence of ADA is challenging. Retrospective analyses have documented such connections in certain case studies. For instance, in a Phase III clinical trial, the engineered factor VIIa analog, vatreptacog alpha, induced ADAs in 11% of hemophilia patients. In contrast, its predecessor, the wild-type recombinant FVIIa (NovoSeven), showed no immunogenicity issues despite over two decades of use in a general population (50). *Post-hoc* analysis revealed that two of the three amino acid substitutions in vatreptacog alpha created novel T-cell epitopes. These epitopes exhibited high-affinity binding to specific HLA-DRB1 receptors, as confirmed by an ex vivo T-cell activation assay, aligning with the observed clinical outcomes. In another example, LMB-100, an antibody-toxin conjugate consisting of a humanized anti-mesothelin Fab linked to a segment of Pseudomonas exotoxin A, demonstrated significantly reduced immunogenicity in a Phase I clinical trial. This trial involved patients with mesothelioma and other solid tumors expressing mesothelin. The decrease in immunogenicity, compared to its wild-type counterpart SS1P, was primarily attributed to the systematic removal of B- and T-cell epitopes (51). Nevertheless, the multifactorial nature of immunogenicity—encompassing treatment modality, product formulation, and patient-related factors—may currently limit the ability to adopt a rational and systematic approach to effectively minimize immunogenicity risks in the target population, unless additional measures are implemented (45).

While immune-related findings must be carefully evaluated in terms of their clinical implications and impact on patient safety, the vast majority of marketed biotherapeutics exhibit anti-drug antibody (ADA) rates below 20% (52). These rates can vary significantly depending on the medical indication and the specific patient context. These findings specifically highlight that most treated patients do not experience severe adverse immune reactions and suggest that conducting a thorough analysis of patient samples could facilitate a more systematic investigation into the root causes of immunogenicity, particularly in cases where ADAs result from a T cell-dependent activation pathway. For instance, a genetic analysis of 1240 treatment-naïve patients from a multicenter, UK-wide prospective observational cohort, who were treated with Infliximab or Adalimumab for Crohn’s disease, revealed that carriers of the HLA-DQA*05 allele, present in approximately 40% of Europeans, were at a higher risk of developing ADAs compared to non-carriers (53).

In this manuscript, we present a novel clinical research tool, termed “clinical MAPPs,” designed to profile potential T cell epitopes directly in patients prior to receiving a biotherapeutic. A key advantage of this approach, particularly if applied systematically throughout clinical trials, is its potential to directly link the presentation of potential T cell epitopes by antigen-presenting cells (APCs) to ADA incidence in treated (and genotyped) patients. This could provide the missing connection between a cause, specifically, the presentation of an MHC-II peptide derived from the biotherapeutic, and its clinical consequence. The clinical MAPPs assay introduced here builds upon the principles and general methodology of the MAPPs assay we have previously described (22, 23). We have continued to optimize and refine the sample preparation protocol to enhance its robustness and throughput, achieving several key improvements: (1) a reduction in inter-operator variability and operational errors, thereby improving reproducibility; (2) a significant decrease in sample processing time; and (3) the ability to simultaneously process up to 96 samples, compared to only 24 in the previous protocol. Combined with the use of a new generation of mass spectrometers, we demonstrate that the clinical MAPPs protocol offers sufficient sensitivity to identify HLA-DR receptor-specific, compound-derived peptides from as few as 350000 monocyte-derived dendritic cells (moDCs) originating from CD14+ monocytes.

Importantly, we also established that the MAPPs assay produces reproducible results regardless of whether monocytes are isolated from fresh or frozen peripheral blood mononuclear cells (PBMCs). Using KLH (keyhole limpet hemocyanin) as a positive control, we confirmed that the assay yields highly comparable results in terms of the number of peptides and clusters presented by moDCs derived from either source. Interestingly, we observed a higher average number of KLH-derived clusters in moDCs generated from frozen PBMCs, which may be due to increased internalization of the exogenous compound, thereby enhancing MHC-II presentation. To account for variability in cell viability and recovery, we recommend starting with 1.35 million CD14+ monocytes (equivalent to approximately 8.5 mL of whole blood or ~9 million PBMCs), as this empirically provided the most robust cluster identification profiles. In summary, the updated protocol enables the Clinical MAPPs assay to be performed on a 10mL blood sample—a reasonable volume for patient-derived samples—and is fully compatible with the use of frozen PBMCs. This flexibility makes the protocol well-suited for processing and analyzing samples collected from patients enrolled in clinical trials.

In our manuscript, we also examine and discuss the advantages of using moDCs as a suitable surrogate for blood-derived DCs in the MAPPs assay. DCs are a rare population of mononuclear cells in blood, comprising only about 1% of all peripheral blood PBMCs (54). Even with our current, more sensitive clinical MAPPs protocol, a blood volume of 200 mL is required to isolate enough DCs for a single condition. Therefore, generating DCs *in vitro* from the more abundant blood monocytes, which make up approximately 15% of PBMCs, offers a practical and efficient alternative (39). This is achieved by stimulating monocytes with a combination of granulocyte-macrophage colony-stimulating factor (GM-CSF) and interleukin-4 (IL-4) cytokines (42, 54–58). The phenotypic similarity between moDCs and blood-derived DCs—including shared characteristics such as dendritic morphology and antigen presentation function (54, 59, 60)—has led to their widespread use as surrogates in MAPPs workflows (42, 54, 61). However, the comparability of activation marker expression and MHC-II peptide presentation between moDCs and blood-derived DCs remains largely unexplored (21). For the first time, we demonstrate that these two cell populations isolated from the same donor exhibit high expression levels of HLA-DR, CD40, CD80, and CD86, with CD83 expression increasing progressively over the activation time course. This observation aligns with previous findings on DCs (40, 62) and moDCs (44, 63, 64) marker expression reported in separate studies. In blood-derived DCs, the expression of CD83, CD86, and HLA-DR peaked approximately 18 hours after stimulation, whereas in moDCs, the activation marker kinetics, particularly HLA-DR expression, were flatter and more sustained, persisting even at 24 hours post-stimulation. In the MAPPs assay, both moDCs and blood-derived DCs from the same donor presented overlapping MHC-II peptides and clusters. However, we observed that the optimal peptide presentation time point for moDCs was 24 hours post-stimulation in 2 out of 3 donors. This discrepancy between the two cell types may reflect differences in the kinetics of overall HLA-DR surface expression compared to the peptide processing and presentation dynamics for exogenously administered drugs (65). In the context of the assay, it is likely that moDCs do not mature as uniformly as blood-derived DCs, which may result in a sustained production of neo-MHC-II receptor-peptide complexes over time and explain why the optimal stimulation time for moDCs is 24 hours. Overall, while moDCs are not strictly identical to blood-derived DCs, we conclude that they serve as an acceptable surrogate for use in the MAPPs assay. This conclusion is further supported by the fact that the *ex vivo* assay is not intended to fully replicate the *in vivo* biological environment.

In this study, we describe a generic clinical MAPPs analysis using PBMC samples isolated from patients enrolled in a clinical trial, demonstrating the feasibility and potential utility of this approach in a clinical setting. In this pivotal proof-of-concept experiment, we challenged moDCs derived from both patients and healthy donors with the same biotherapeutic and compared the resulting MHC-II peptide profiles between the two groups (66). In this clinical MAPPs experiment, we employed a pan-HLA profiling approach (23) to identify MHC-II peptides potentially binding across the entire HLA receptor repertoire. Unlike preclinical screening strategies, which typically focus on a relatively small number (5-20) of donors (21, 34, 35, 67) to identify major clusters that could impact a large portion of the target population, our goal is to build a comprehensive MHC-II cluster database using data from potentially hundreds of patients. This database could eventually be correlated with individual patient ADA rates and characteristics. To achieve this, it was critical to demonstrate that such an approach could be implemented within the standard sample collection and processing framework of a clinical trial, ensuring routine sample availability. Additionally, we showed that our clinical MAPPs protocol is highly reproducible and quantitative. Importantly, the results obtained from clinical donors were consistent in quality with those derived from a healthy control cohort.

**Figure 7:**
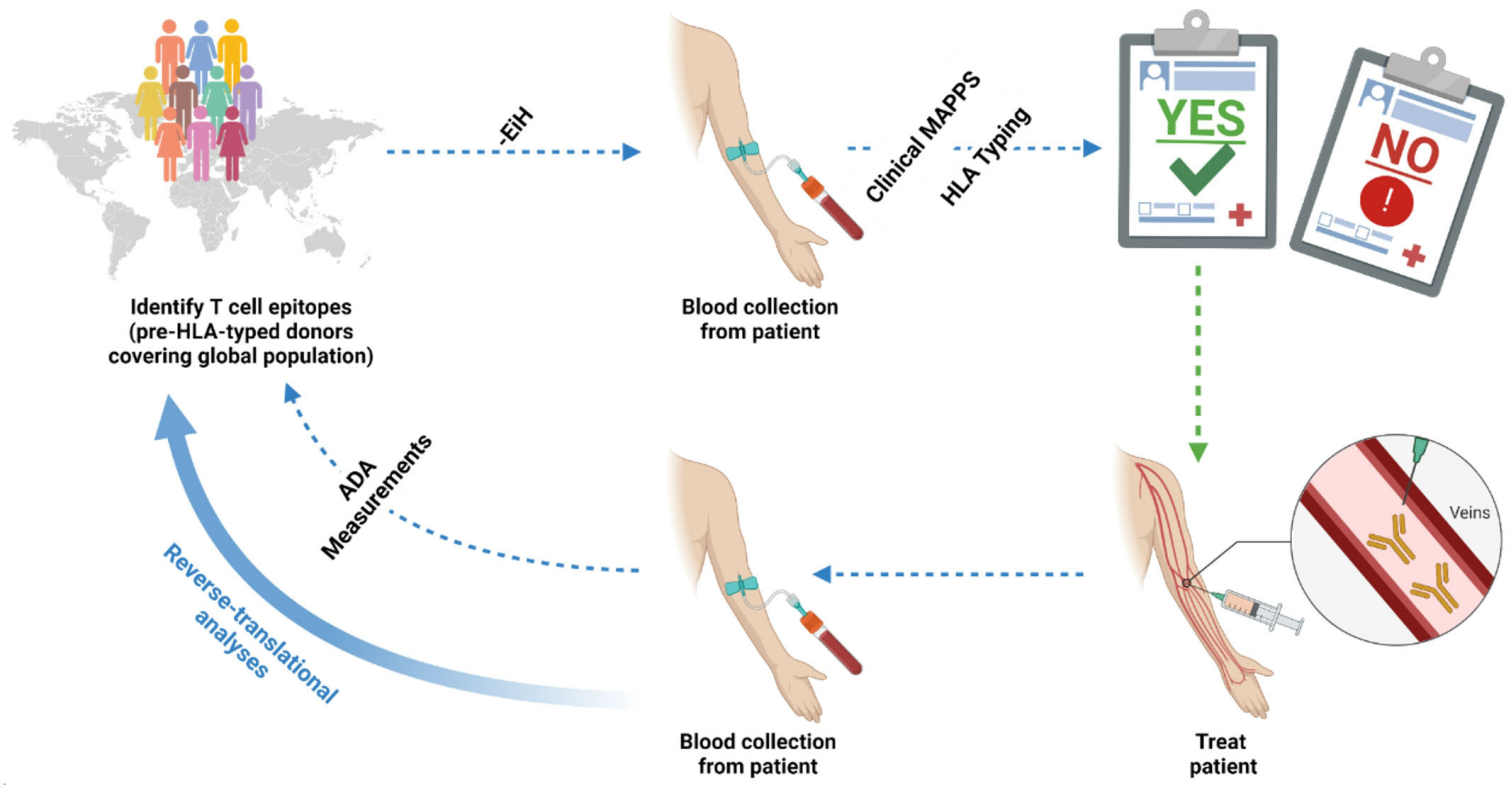
Premise of the clinical MAPPs assay strategy for personalized healthcare. The sequential practice of the clinical MAPPs assay entails, 1) identifying all potential T cell epitopes of the therapeutic antibody via the MAPPs assay by using blood samples from pre-HLA-typed healthy donors that cover the global population, 2) upon entry of the therapeutic antibody into phase I of clinical trials, blood samples are collected from HLA-typed patients, and the clinical MAPPs assay is performed to identify potential T cell epitopes prior to treatment, 3) depending on the outcome of the clinical MAPPs assay, the therapeutic antibody is either administered to the patient or other risk mitigation strategies are carried out, and 4) if the patient received therapeutic antibody treatment, patient blood samples are collected for ADA measurements for reverse translational analysis. EiH, entry into human phase of drug development. Created with BioRender.com.

In summary, we believe the novel clinical MAPPs assay represents a significant advancement in personalized healthcare, with the potential to predict and mitigate immunogenicity risks in clinical settings as relevant data is being measured over time (**Figure 7**). During the early stages of a biotherapeutic’s clinical development, the application of a clinical MAPPs assay to all enrolled patients prior to their first treatment could provide an initial personalized catalogue of potential T cell epitopes (MHC-II peptides derived from the biotherapeutic) associated with an elevated risk of immunogenicity. As clinical trials progress and more data becomes available, such as individualized ADA measurements and potentially results from T cell activation assays (32) to confirm causal relationships, clinical MAPPs could be further utilized to refine patient selection. This would allow healthcare providers to identify patients at higher risk of immunogenicity and select treatments with an improved benefit-risk profile. Ultimately, the cumulative data collected throughout this process, including MAPPs assay results, could contribute to the development of safer, next-generation therapeutics designed for more targeted patient populations. We envision the clinical MAPPs assay as a crucial tool at the intersection of preclinical and clinical risk assessment, enabling the collection of personalized patient data to inform and advance both therapeutic safety and efficacy.

## Data Availability

The datasets presented in this study can be found in the following online repositories: PRIDE archive (https:proteomecentral.proteomexchange.org/cgi/GetDataset?ID=PXD065933), the MassIVE partner repository (MassIVE dataset identifier: MSV000098460).

## Supplementary material

Supplementary Table 1 Adalimumab clusters identified in this manuscript.

Supplementary Table 2 Total unique and adalimumab-specific peptide counts obtained from increasing cell numbers challenged with adalimumab and subjected to an automated or manual immunoprecipitation protocol in the major histocompatibility complex (MHC)-II associated peptide proteomics (MAPPs) assay.

Supplementary Table 4 Characterization of PBMCs isolated either from a healthy or from a clinical cohort. *0.1x10^6^ was the LLOD of the cell counter. Three donors’ preparations were below that threshold.

Supplementary Figure 1 Percent recoveries of fresh versus cryopreserved peripheral blood mononuclear cells (PBMCs). Box plots depicting the percent recovery rates of **(A)** thawed PBMCs, **(B)** monocytes from fresh and frozen PBMCs, and **(C)** compound-challenged/LPS-stimulated monocyte-derived dendritic cells (moDCs) from plated monocytes. Fresh and cryopreserved PBMCs obtained from the same 10 healthy buffy-coat donors. The percent mean is labeled. Each uncolored circle resembles an individual donor.

Supplementary Figure 2 Histogram **(A)** and box plot **(B)** analysis of all peptides analyzed in the MAPPs analysis of KLH in fresh and frozen PBMCs isolated from 10 genotyped donors. In average, more peptides are isolated and identified in a MAPPs analysis using DCs generated from freshly isolated PBMCs. As highlighted in **(A)**, however, the MHC-II peptide length distribution remains very similar independently of the PBMC pre-treatment (fresh or frozen), with a majority of MHC-II peptides exhibiting a length of 15-16 amino acids. Conversely, as delineated in **(B)**, the portion of bona fide MHC-II peptide binders is slightly lower in preparations using frozen PBMC than using fresh PBMCs. In parallel, the portion of “non-binding” peptides is substantially higher in preparations using frozen PBMCs. All data are available from **Supplementary Table 3**.

Supplementary Figure 3 Percent recovery of CD14^+^ monocytes and blood dendritic cells (DCs) isolated from peripheral blood mononuclear cells (PBMCs). Box plots depicting the recovery rates of **(A)** CD14^+^ monocytes and **(B)** blood dendritic cells isolated from PBMCs obtained from the same 29 healthy buffy-coat donors. The mean is denoted as the labeled blue icon, where a mean percent recovery of 15.39% monocytes corresponds to 81 million cells and 0.46% DC recovery corresponds to 2.44 million cells. Each uncolored circle resembles an individual donor.
