# Supplementary figures and images for: Clinical MAPPs: a personalized healthcare-driven assay for the direct identification of potential T cell epitopes in patients
Source: Front Immunol. 2026 Jul 7;17:1879253. doi: 10.3389/fimmu.2026.1879253 (PMC13385703; doi:10.3389/fimmu.2026.1879253)

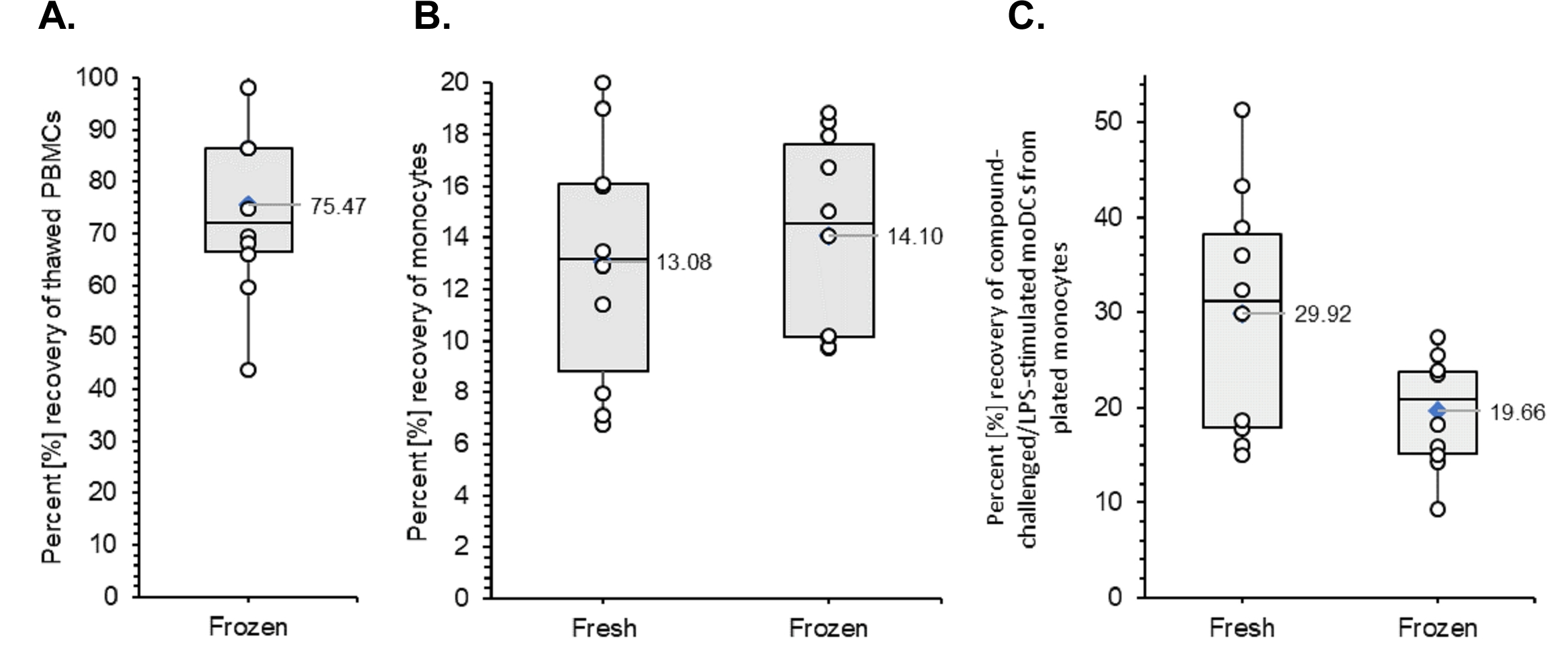

Supplement: Supplementary Figure 1 — Percent recoveries of fresh versus cryopreserved peripheral blood mononuclear cells (PBMCs). Box plots depicting the percent recovery rates of (A) thawed PBMCs, (B) monocytes from fresh and frozen PBMCs, and (C) compound-challenged/LPS-stimulated monocyte-derived dendritic cells (moDCs) from plated monocytes. Fresh and cryopreserved PBMCs obtained from the same 10 healthy buffy-coat donors. The percent mean is labeled. Each uncolored circle resembles an individual donor. [file Image1.jpeg]

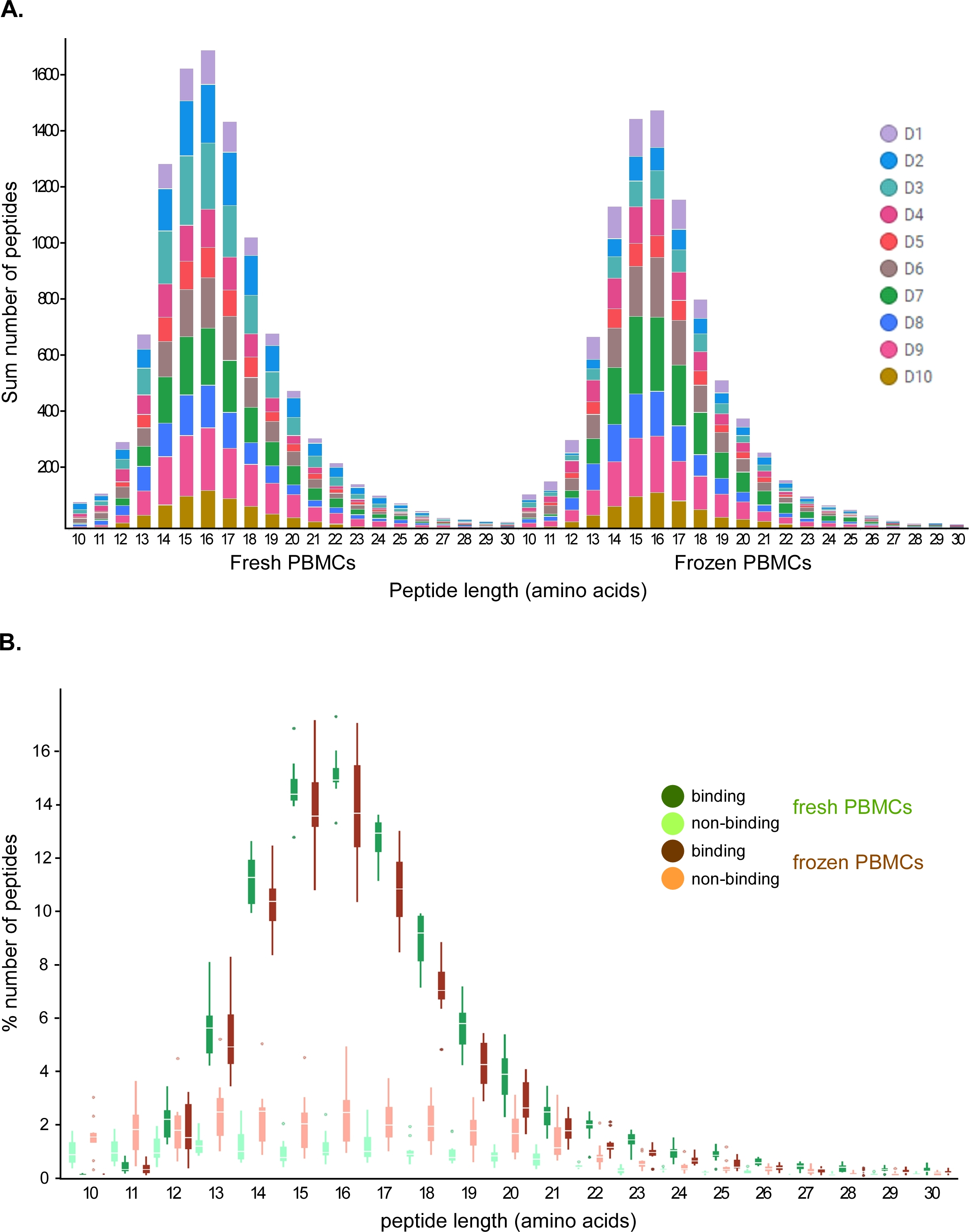

Supplement: Supplementary Figure 2 — Histogram (A) and box plot (B) analysis of all peptides analyzed in the MAPPs analysis of KLH in fresh and frozen PBMCs isolated from 10 genotyped donors. In average, more peptides are isolated and identified in a MAPPs analysis using DCs generated from freshly isolated PBMCs. As highlighted in (A), however, the MHC-II peptide length distribution remains very similar independently of the PBMC pre-treatment (fresh or frozen), with a majority of MHC-II peptides exhibiting a length of 15-16 amino acids. Conversely, as delineated in (B), the portion of bona fide MHC-II peptide binders is slightly lower in preparations using frozen PBMC than using fresh PBMCs. In parallel, the portion of “non-binding” peptides is substantially higher in preparations using frozen PBMCs. All data are available from Supplementary Table 3. [file Image2.jpeg]

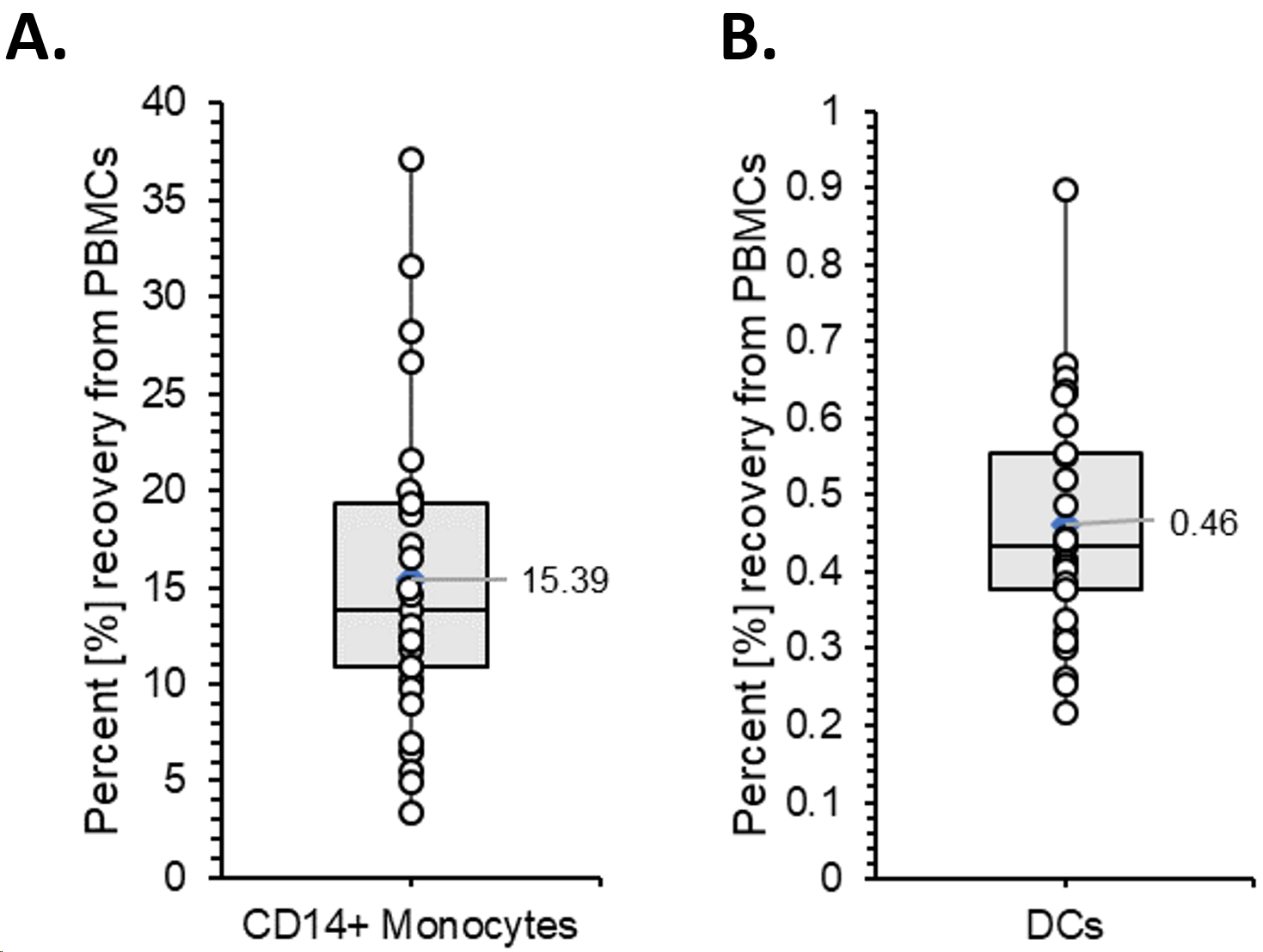

Supplement: Supplementary Figure 3 — Percent recovery of CD14+ monocytes and blood dendritic cells (DCs) isolated from peripheral blood mononuclear cells (PBMCs). Box plots depicting the recovery rates of (A) CD14+ monocytes and (B) blood dendritic cells isolated from PBMCs obtained from the same 29 healthy buffy-coat donors. The mean is denoted as the labeled blue icon, where a mean percent recovery of 15.39% monocytes corresponds to 81 million cells and 0.46% DC recovery corresponds to 2.44 million cells. Each uncolored circle resembles an individual donor. [file Image3.jpeg]
